# Stimulated phospholipid synthesis is key for hepatitis B virus replications

**DOI:** 10.1038/s41598-019-49367-8

**Published:** 2019-09-10

**Authors:** Qingxia Huang, Hehua Lei, Laifeng Ding, Yulan Wang

**Affiliations:** 1 State Key Laboratory of Magnetic Resonance and Atomic and Molecular Physics, National Center for Magnetic Resonance in Wuhan, Key Laboratory of Magnetic Resonance in Biological Systems, Wuhan Institute of Physics and Mathematics, Chinese Academy of Sciences, Wuhan, 430071 P.R. China; 2 University of Chinese Academy of Sciences, Beijing, 100049 P.R. China; 3 Singapore Phenome Center, Lee Kong Chian School of Medicine, Nanyang Technological University, 59 Nanyang Drive, Singapore

**Keywords:** Metabolomics, Viral infection

## Abstract

Chronic hepatitis B Virus (HBV) infection has high morbidity, high pathogenicity and unclear pathogenesis. To elucidate the relationship between HBV replication and host phospholipid metabolites, we measured 10 classes of phospholipids in serum of HBV infected patients and cells using ultra performance liquid chromatograph-triple quadruple mass spectrometry. We found that the levels of phosphatidylcholine (PC), phosphatidylethanolamine, and lyso-phosphatidic acid were increased in HBsAg (+) serum of infected patients compared with HBsAg (−), while phosphatidylserine, phosphatidylglycerol, phosphatidylinositol, and sphingomyelin were decreased, which were confirmed in an HBV infected HepG2.2.15 cell line. We further evaluated the enzyme levels of PC pathways and found that PCYT1A and LPP1 for PC synthesis were up-regulated after HBV infection. Moreover, HBV replication was inhibited when PCYT1A and LPP1 were inhibited. These results indicated that the PC synthesis in HBV infected host are regulated by PCYT1A and LPP1, which suggests that PCYT1A, LPP1 could be new potential targets for HBV treatment.

## Introduction

Hepatitis B Virus (HBV) is a double stranded DNA virus and belongs to hepadnavirdae family. HBV infection causes a severe liver infectious disease and has become a global problem affecting human health. In 2015, WHO estimated that approximately 257 million people, equivalent to 3.5% of the population, were infected with chronic HBV worldwide, resulting in 887, 000 HBV-related deaths annually^1^. Many liver conditions are associated with HBV infection, which include acute hepatitis, chronic hepatitis B (CHB), cirrhosis and hepatocellular carcinoma (HCC)^2,3^. HCC associated with HBV infection is considered as an arduous scientific mission^4–6^, as HBV infection attributes to over 50% of HCC cases worldwide and up to 65% cases in China^3,7^. However, the mechanism of HCC associated with HBV remains elusive. The current theory is that HBV DNA integrates into the host genome and continues expression of viral proteins such as HBx and large envelope protein (LHBs), causing oxidative stress and genetic instability, eventually leading to hepatocyte transformation and HCC^8^. Ganem *et al*. reported that the carcinogenesis of chronic HBV infection is related to inflammation^9^. The current clinical strategy aims at inhibiting HBV replication^10^. However, despite the effectiveness of hepatitis B vaccination, there is no effective cure for HBV infection. Therefore, it is necessary to investigate the complex host metabolic response to HBV replication and provide information for further our understanding of the pathogenesis of HBV infection, which would be vital for discovering of new potential therapeutic targets for combatting HBV infection.

Previously, investigation of metabolic response of HBV infection revealed that HBV infection induces stimulated hexosamine metabolic pathways, and inhibiting key enzymes, glutamine-fructose-6-phosphateamidotransferase 1 (GFAT1), with 6-diazo-5-oxo-L-norleucine (DON) able to suppress HBV replication and expression of viral proteins^11^. DON is also known to suppress cancer growth; nevertheless, it failed in clinical trials due to its toxicity^12,13^. In the same investigation, we also found that stimulated phosphatidylcholine *via* choline kinase alpha (CHKA) is associated with HBV infection and inhibiting CHKA can also suppress HBV replication^11^. This suggests that phospholipids could play an important role in HBV replication. Phospholipids are known to be the main constituents of cell membrane and the biological function of phospholipid species have been recognized recently^14,15^. Naguib *et al*. have shown that a p53 mutation could change the acyl chain composition of phosphatidylinositol^16^, while Louie *et al*. reported that cancer cells incorporate and remodel exogenous palmitate into oncogenic signaling lipids such as glycerophospholipids and sphingolipids^17^. In addition, HBV virus contains an envelope, in which more than 90% are composed of phospholipids, with the lipids of hepatitis B surface antigen (HBsAg) obtained from the host cell membrane^18^. Several studies demonstrated that HBV infection induced changes in the composition of phosphatidylcholines in HBV-infected mouse livers^19^. Huang *et al*. identified a set of chronic hepatitis B (CHB)-associated biomarkers including lyso-phosphatidylcholines, phosphatidylcholines, phosphatidylinositol, phosphatidylserine in CHB patients^20^. These reports emphasized important roles of phospholipids in HBV infection.

In this study, we analyzed 10 classes of major phospholipids in an HBV infected patient’s serum and HBV infected cells by targeted ultrahigh-performance liquid chromatography system coupled to a triple-quadrupole mass spectrometer (UHPLC-MS) analysis and these included phosphatidic acid (PA), phosphatidylcholine (PC), phosphatidylethanolamine(PE), phosphatidylglycerol (PG), phosphatidylinositol (PI), phosphatidylserine (PS), sphingomyelin (SM), lyso-phosphatidic acid (LPA), lyso-phosphatidylcholines (LPC) and lyso-phosphatidylethanolamine (LPE). We identified metabolic pathways that were altered by HBV infection. We further validated that the phospholipids metabolic pathways played important roles in HBV replication. Our investigation provided more information furthering the understanding of the pathogenesis and potential new targets of HBV treatment.

## Results

### Phosphatidylcholine is increased in serum from HBsAg (+) participants

The clinical characteristics of HBsAg (−) and HBsAg (+) patients are showed in Table 1. 60% of the HBsAg (−) group (n = 48) were males, while 62% of the HBsAg (+) group (n = 40) were males. The age of the population selected was matched. In addition, there were no significant differences in the levels of ALT, AST, triglyceride, HDL, LDL between HBsAg (−) and HBsAg (+) groups. These results indicated that the demographic characteristics of HBsAg (−) and HBsAg (+) groups were well matched.

**Table 1:** The clinical characteristics of patients.

Characteristic	HBsAg (−)	HBsAg (+)	*p* value^a^
Male (n, %)	29 (60.42%)	25 (62.50%)	0.842^b^
Female (n, %)	19 (39.58%)	15 (37.50%)	
Age (years)	54.63 ± 10.18	58.63 ± 9.28	0.059
ALT (U/L)	22.30 ± 15.75	24.84 ± 15.20	0.454
AST (U/L)	25.60 ± 16.48	30.21 ± 19.39	0.243
Triglyceride (mmol/L)	1.78 ± 1.27	1.44 ± 0.76	0.137
HDL (mmol/L)	1.14 ± 0.29	1.11 ± 0.33	0.636
LDL (mmol/L)	2.39 ± 0.79	2.11 ± 0.59	0.071

Data are shown as numbers (percentage) or mean ± SD, n [HBsAg (−)] = 48, n [HBsAg (+)] = 40. ^a^t-test; ^b^Chi-Square test; *p* value < 0.05 considered to be statistically significant. ALT: alanine aminotransferase, AST: aspartate aminotransferase, HDL: high density lipoprotein, LDL: low density lipoprotein.

**Figure 1:**
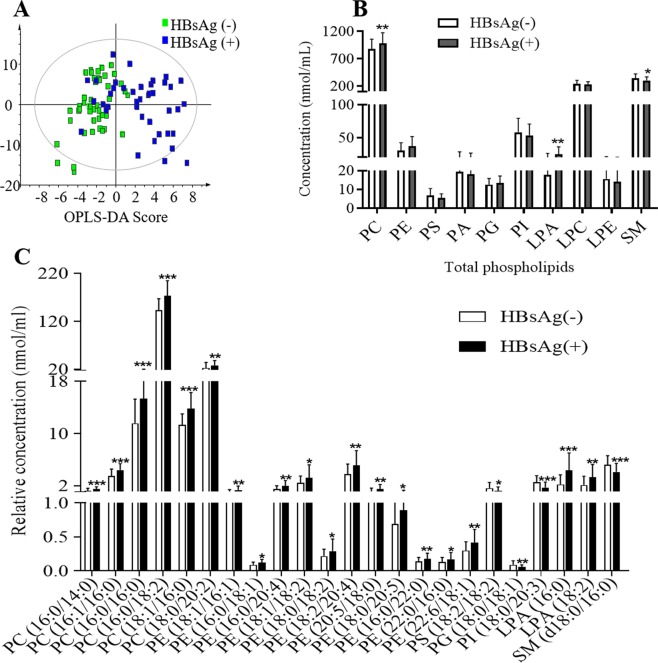
Phospholipids analysis of HBsAg (−) and HBsAg (+) participants. (**A**) OPLS-DA scores plots showed the separation between HBsAg (−) and HBsAg (+) patients. Q^2^ = 0.444, *p* = 5.02 × 10^−10^ (CV-ANOVA), Q^2^ > 0.4 is considered to be a good predictor for the model, and *p* value < 0.05 indicates the validity of the model. (**B**) The total amount of each class of phospholipid in human serum. (**C**) The significant difference of phospholipids between HBsAg (−) and HBsAg (+) participants. The significance of the metabolites was selected with the criteria that satisfied the followings: variable importance in the projection (VIP) > 1 (Fig. S2A), *p* value < 0.05, and fold change > 1.2 or < 0.8 (Fig. S2B). The histogram is expressed as mean ± SD, n [HBsAg (−)] = 48, n [HBsAg (+)] = 40, detailed data of phospholipids are shown in Table S1. ***p value < 0.001, **p value < 0.01, *p value < 0.05. PC: phosphatidylcholine, PE: phosphatidylethanolamine, PS: phosphatidylserine, PG: phosphatidylglycerol, PI: phosphatidylinositol, LPA: lyso-phosphatidic acid, LPE: lyso-phosphatidylethanolamine, SM: sphingomyelin.

In this study, 131 phospholipid species were quantified in human serum, including 46 PCs, 22 PEs, 4 PSs, 2 PAs, 5 PGs, 14PIs, 6 LPAs, 13 LPCs, 6 LPEs and 13 SMs (Table S1 and Supplementary dataset 1). The OPLS-DA analysis showed that phospholipid profiles of serum with HBsAg (−) and HBsAg (+) groups were well separated (Fig. 1A). In addition, The total levels of PC and LPA increased while the total levels of SM decreased in HBsAg(+) group comparing to the HBsAg (−) group (Fig. 1B). The total levels of 23 phospholipid species were significantly changed in HBsAg (+) group comparing to the HBsAg (−) group (Fig. 1C). These changed phospholipids were selected based on parameters generated from multivariate data analysis: VIP > 1 (Fig. S1A) and *p* value < 0.05, and in the meantime satisfying the condition of fold change >1.2 or <0.8 (Fig. S1B). The levels of PCs, PEs and LPAs were increased while the levels of PSs, PGs, PIs and SMs were decreased in serum of HBsAg (+) participants compared to those of HBsAg (−) participants (Fig. 1C). The receiver operating characteristic (ROC) analysis of 23 phospholipid species (Table S2) found that 9 phospholipid species including PC (18:1/16:0), PC (16:0/18:2), PC (16:0/16:0), PC (16:1/16:0), PC (16:0/14:0), PE (22:6/18:1), PE (16:0/20:4), PE (18:2/20:4), LPA (16:0) had greater diagnostic power with an area under the ROC (AUC) greater than 0.7, and the predictive power of PC were greater than PE and LPA (Table 2). These findings suggest that the up-regulation of PC is strongly related to HBV replication.

**Table 2:** ROC analysis of serum and AUC Results.

Phospholipids	AUC
PC (18:1/16:0)	0.799
PC (16:0/18:2)	0.777
PC (16:0/16:0)	0.759
PC (16:1/16:0)	0.752
PC (16:0/14:0)	0.733
PE (22:6/18:1)	0.715
PE (16:0/20:4)	0.707
PE (18:2/20:4)	0.700
LPA (16:0)	0.756

ROC: Receiver-operating characteristic;

AUC: Area under the receiver-operating characteristic.

### Phosphatidylcholine analysis of HepG2 and HepG2.2.15 cells

**Figure 2:**
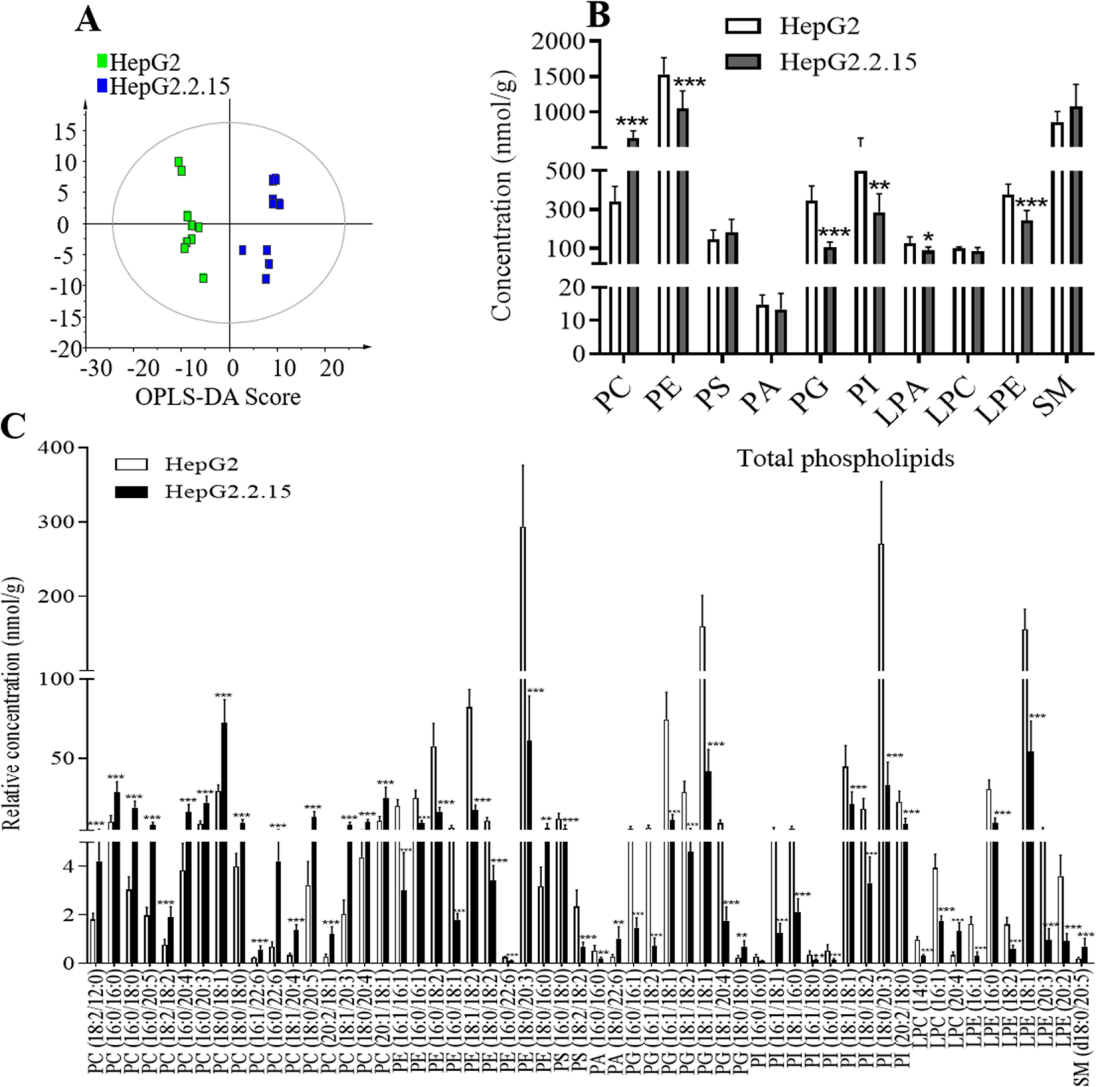
Phospholipids analysis of HepG2 and HepG2.2.15 cells. (**A**) OPLS-DA scores plots showing the separation between HepG2 and HepG2.2.15 cells, Q^2^ = 0.902, *p* = 1.90 × 10^−6^. (**B**) The total amount of each class of phospholipid in cells. (**C**) The significant difference of phospholipids between HepG2 and HepG2.2.15 cells. The significance of the metabolites was used the criteria that variable VIP > 1, *p* value < 0.05, and fold change > 1.2 or < 0.8. The histogram is expressed as mean ± SD, n = 10, detailed data of phosphatidylcholines are shown in Table S3. ***p value < 0.001, **p value < 0.01, *p value < 0.05.

In order to confirm the association between the HBV infection and the up-regulation of PC in the host, cell models were used to further analyze the changes of PC in host cells infected with HBV. HepG2.2.15 cell lines were obtained from the human hepatoblastoma cell lines HepG2 by transfection of HBV DNA, which are widely used in the study of HBV infection due to its stable expression and replication of HBV^21,22^. In this study, a total of 162 phospholipid species were quantified in HepG2 and HepG2.2.15 cells (Table S3 and Supplementary dataset 2), and among these, 56 phospholipid species were significantly different between HepG2 and HepG2.2.15 cells (Fig. 2). The total levels of PC were increased in HepG2.2.15 cells comparing to HepG2 cells, and those of PE, PG, PI, LPA and LPE were decreased in HepG2.2.15 cells (Fig. 2B). Levels of all PCs were increased in HepG2.2.15 cells, whereas levels of PSs, PGs and PIs were decreased in HepG2.2.15 cells (Fig. 2C), which are consistent with the results obtained from HBV infected human populations. Compared with the control group, both PEs and SMs were different between the serum of HBsAg (+) participants and HepG2.2.15 cells, however, the trend of change was not consistent. LPAs were found to increase only in the serum of HBsAg (+) and LPEs were found to decrease only in cell models. These findings again suggested a strong association between up-regulation of PC in host and HBV infection.

### Up-regulation of the expression of PCYT1A and LPP1 contributes to the increase of phosphatidylcholine

**Figure 3:**
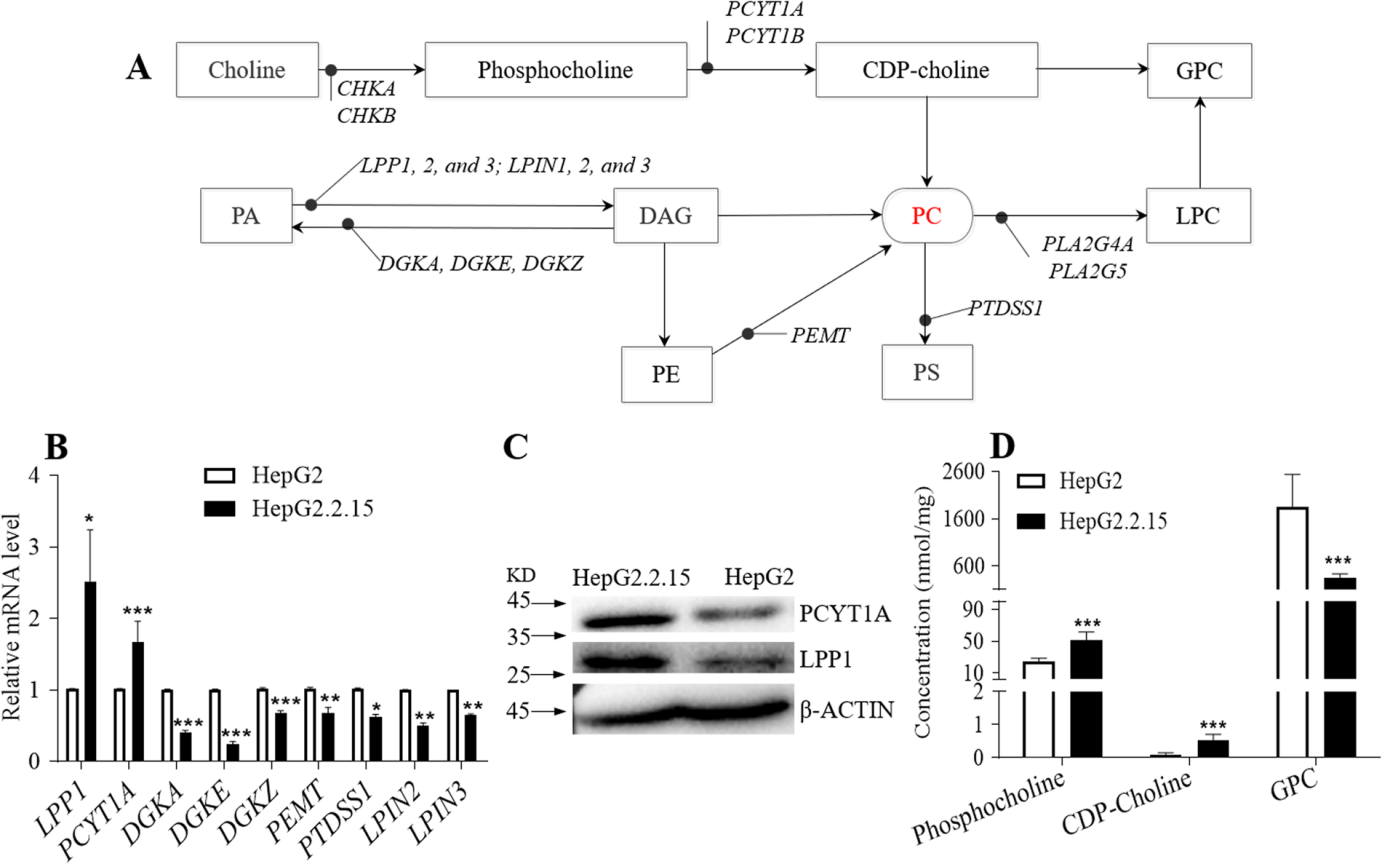
The relative mRNA expression levels of major genes in PC synthesis and metabolic pathways. (**A**) Phosphatidylcholine synthesis and metabolism pathways. (**B**) The relative mRNA level of major genes in phosphatidylcholine synthesis and metabolism in HepG2 and HepG2.2.15 cells, n = 3, detailed data are shown in Table S4. (**C**) The western blot analysis of LPP1, PCYT1A protein expression levels in HepG2 and HepG2.2.15 cells, the initial figure is shown in Fig. S2. (**D**) The levels of the substrates of the PC synthesis in HepG2 and HepG2.2.15 cells, n = 10. (**B**,**D**) data are shown as mean ± SD, t-test, ***p value < 0.001, **p value < 0.01, *p value < 0.05. PA: phos*p*hatidic acid, DAG: diglyceride, PC: phosphatidylcholine, PE: phosphatidylethanolamine, PS: phosphatidylserine, LPC: lyso-phosphatidylcholine, GPC: glycerol-phosphorylcholine.

PC is mainly synthesized by two pathways, the CDP-choline pathway (Kennedy pathway) and phosphatidylethanolamine N-methyltransferase (PEMT) pathways (Fig. 3A)^23,24^. The substrates diacylglycerol (DAG) and CDP-choline required for PC synthesis come from the metabolism of PA and choline, respectively. The metabolism of PC mainly produces LPC and fatty acids or is used to synthesizes PS(Fig. 3A)^25,26^. To validate the association between up-regulation of PC and HBV infection further, we examined the relative mRNA expression levels of major genes in PC synthesis and metabolic pathways (Table S4).The results revealed that the expression of *PCYT1A* and *LPP1* were significantly up-regulated in HepG2.2.15 cells, and the relative mRNA levels of *LPIN2, LPIN3, DGKA, DGKE, DGKZ* and *PEMT* were down-regulated (Fig. 3B). In addition, we confirmed that the levels of LPP1 and PCYT1A in HepG2.2.15 cells were enhanced compared with HepG2 cells (Figs 3C and S2). These findings showed that HBV infection could promote PC synthesis by up-regulating the levels of PCYT1A and LPP1. Furthermore, we also measured the levels of the substrates produced during PC synthesis (choline, phosphocholine, CDP-choline and GPC) in both HepG2 and HepG2.2.15 cells. We found that the levels of phosphocholine and CDP-choline increased and the levels of GPC decreased in HepG2.2.15 cells compared to HepG2 cells (Fig. 3D). These results confirmed that the increase of PC level is due to up-regulation of PCYT1A and LPP1, and is characteristic of HBV infection.

### Down-regulations of PCYT1A and LPP1 inhibits HBV replication

**Figure 4:**
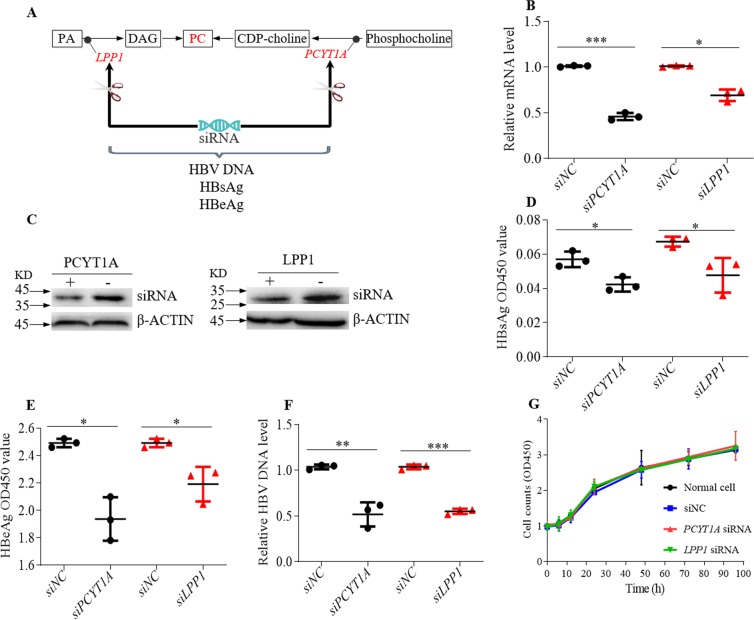
HepG2.2.15 cells were transfected by siRNA. (**A**) The framework of gene silencing with siRNA. (**B**) The relative mRNA levels of *PCYT1A* and *LPP1*. (**C**) The western blot analysis of LPP1, PCYT1A protein expression levels in the siRNA negative control group and treatment group, the initial figure is shown in Fig. S3. (**D**) The relative levels of HBsAg. (**E**) The relative levels of HBeAg. (**F**) The relative levels of HBV DNA. (**G**) The proliferation of HepG2.2.15 cells transfected by siRNA was assayed using cell-counting assay. (**B**,**D**–**F**) data are shown as mean ± SD, n = 3, t-test ***p value < 0.001, **p value < 0.01, *p value < 0.05. PA: phos*p*hatidic acid, DAG: diglyceride, PC: phosphatidylcholine. siRNA: si*PCYT1A* or si*LPP1*, siNC: negative control siRNA.

One emerging treatment strategy for chronic HBV is RNA interference (RNAi), which is a process of inhibiting gene expression by inducing gene silencing at the post-transcriptional level through small interfering RNA (siRNA) molecules^27–30^. In order to study the impact of PCYT1A and LPP1 on the HBV replication, we suppressed the expression of *PCYT1A* and *LPP1* by transfection HepG2.2.15 cells with siRNAs (Figs 4B,C and S3). The results showed that down-regulation of both PCYT1A and LPP1 led to a significant reduction in the levels of HBV DNA replications (Fig. 4F). In addition, the levels of HBsAg and HBeAg also decreased (Fig. 4D,E). These results suggested that down-regulation of PCYT1A and LPP1 inhibited HBV replication. We further demonstrated that the reduced levels of HBV replication were not due to cell growth. This was confirmed by comparing the viable cells treated with the siRNA and siRNA negative control group and normal cell group (Fig. 4G). The results demonstrated that down-regulation of PCYT1A and LPP1 expression *via* siRNA interference is capable of inhibiting the HBV replication.

## Discussion

HBV infection is a chronic infectious disease, which contributes to 50–60% cases of liver cancer^3,7^. Although vaccination is effective, there is still no drug that can eliminate the virus completely. The current treatment strategy is to inhibit HBV replication^10^. HBV virus is known to integrate host phospholipids to form hepatitis B surface antigen^18^. Previously, we demonstrated that HBV infection caused up-regulation of CHKA and concurrent increases in the levels of phosphatidylcholine^11^.

In this study, we aim to further investigate the changes of phospholipids in both human with positive HBV infection and in HepG2.2.15. In order to do this, we first measured a total of 10 main class of phospholipids in patients and cells. We were able to quantify a total of 131 phospholipid species in human serum (Table S1), in which the levels of PCs, PEs and LPAs increased in HBsAg (+) participants compared with HBsAg (−) participants, while the levels of PSs, PGs, PIs and SMs decreased in which HBV infected participants (Fig. 1B). In addition, the diagnostic power of increased levels of PC was greatest among the altered phospholipid species (Table 2). We subsequently confirmed associations of elevations of PC with HBV infection in HepG2.2.15 cells that is well accepted cell model for investigating HBV infection (Fig. 2B). Our conclusion that HBV infection caused the up-regulation of PC level in host is consistent with our previous investigation on HepG2.2.15 cells^11^ and human CHB patients^20^. However, the total level of PE was only down-regulated in cells, but not changed in human serum. And the changes in PE species were also different between human serum and cells. This may because the level of PE in human blood is regulated by the whole system, resulting in small changes in PE.

It is essential to identify the enzyme contributing to the elevated levels of PC in HBV infection, so that a therapy can be developed to reduce or eliminate HBV virus from hosts. We analyzed the relative mRNA expression levels of major genes in PC synthesis and metabolism (Fig. 3A). We found that the expression levels of PCYT1A and LPP1 were up-regulated in HepG2.2.15 cells (Fig. 3B). PCYT1A is a rate-limiting enzyme for PC synthesized *via* the Kennedy pathway, which converts phosphocholine to CDP-choline^31^. Many studies have reported that the increase of PC synthesis is caused by the up-regulation of PCYT1A^11,19,31,32^. Furthermore, we examined the levels of the substrates for the PC biosynthesis in HepG2 and HepG2.2.15 cells and found that phosphocholine and CDP-choline increased in HepG2.2.15 cells (Fig. 3B). These results demonstrated that the increase of PC is consistent to the up-regulation of PCYT1A. In addition, LPP1 is the second class of phosphatidate phosphohydrolase (PAP-2) in mammalian cells^33^, which catalyzes dephosphorylation of PA to synthesize DAG. DAG is another substrate for synthesizing PC in addition to CDP-choline (Fig. 3A). Studies by Long *et al*. showed that the activity of LPP1 is closely related to signaling lipid molecules, such as DAG, sphingosine-1-phosphate (S1P), LPA, etc^34^. It is reported that overexpression of LPP1 can inhibit the production of inflammatory factor IL-8 and the activity of LPA receptor^35^. The increase in LPP1 activity may also reduce the migration of fibroblast cells by reducing the formation of PA^36^. In the current study, we report for the first time that up-regulation of the expression of LPP1 of host is closely related to up-regulation of PC synthesis in HBV infected host cells, which may imply that LPP1 could play an important role in the pathogenesis of HBV infection.

So far, we have demonstrated that the up-regulated PC levels *via* increased expression of PCYT1A and LPP1 and PC is likely to be essential for HBV virus replication, particularly since more than 80% lipids of the envelope of HBV are phosphatidylcholine^18^, and 65% phospholipids of HBsAg are phosphatidylcholine^37^. The up-regulation of phosphatidylcholine synthesis can provide sufficient lipids for the synthesis of complete HBV envelope. We subsequently inhibited the expression of PCYT1A and LPP1 by transfection siRNA in HepG2.2.15 cells in order to elucidate whether the expression of PCYT1A and LPP1 could affect HBV replication. We found decreased levels of both HBsAg and HBeAg, and HBV DNA, which were used as markers of HBV DNA replication^38^, indicating that the HBV replication was inhibited (Fig. 4). In addition, we confirmed that the siRNA treatment did not affect the HepG2.2.15 cell replication (Fig. 4F), which further validated that inhibiting expressions of PCYT1A and LPP1 can deter HBV replication. These results suggested that both PCYT1A and LPP1 could potentially be new drug targets for HBV treatment.

In summary, we have shown that the increased levels of PC in the HBV infected host are strongly related to HBV replication. In addition, the HBV infection promoted the PC synthesis by up-regulating the expressions of PCYT1A and LPP1 in the host. Furthermore, HBV replication were inhibited by down-regulation of the PCYT1A and LPP1 expressions *via* siRNA interference. These results indicated that both PCYT1A and LPP1 could be potential drug targets for HBV treatment. Our study provided detailed information on the phospholipid changes of host in response to HBV infection, and shed new light on potential drug targets for the treatment of HBV infection.

## Methods

### Patients and cell culture

Wuhan Institute of Physics and Mathematics, Chinese Academy of Sciences approved this study and all participants provided written informed consent prior to inclusion, all the methods were performed in accordance with the relevant guidelines and regulations. A total of 88 serum samples [40 with HBsAg (+) and 48 with HBsAg (−)] were provided from Tongji Hospital, Tongji Medical College, Huazhong University of Science and Technology. The human hepatoblastoma cell line HepG2 and HepG2.2.15 cells (with stable expression and replication of HBV) were purchased from China Center for Type Culture Collection (CCTCC, Wuhan, China). Both HepG2 and HepG2.2.15 cells were cultured in MEM medium with 10% fetal bovine serum at 37 °C and 5% CO_2_. The cells were digested by trypsinization and washed with cold PBS 3 times, then stored at −80 °C until extraction.

### Phospholipids analysis

Phospholipids extraction, identification and quantification were performed as described by our previous publication^39^. In brief, phospholipids were extracted by chloroform^40^. We employed three steps for the qualitative and quantitative analysis of phospholipids using an ultrahigh-performance liquid chromatography system coupled to a triple-quadrupole mass spectrometer (UHPLC-MS). The first step is to pre-scan the precursor ions of phospholipids through MRM with a mixture containing aliquots of all the samples. The second step is to identify the structure of phospholipids through MS/MS spectra performed on only the selected ions that presented in samples by step 1. The third step is to quantitatively analyze the identified phospholipid molecules by MRM. PC, PE, PG, PA, PI and PS were quantified using relative response and internal standards, while LPC, LPE, LPA and SM were quantified using internal standards for relative quantification. The nomenclature of phospholipids is consistent with LIPID MAPS (http://www.lipidmaps.org/).

### UHPLC-MS analysis of choline metabolites

The cells were re-suspended in 500 μL methanol/water solution (v/v, 2:1) and subjected to three free-thaw cycles, followed by sonication in a wet ice bath for 15 min (1 min power following 1 min stop). 100 μL internal standard (200 nM choline-d9) and 400 μL methanol/water solution (v/v, 2:1) were added. The mixture was centrifuged at 11, 060 g for 10 min at room temperature after 30 s of vortex mixing. The top layer was collected and stored at −20 °C before the UHPLC-MS analysis.

The UHPLC-MS system included an Agilent 1290 ultrahigh-performance liquid chromatography (UHPLC) system coupled to a 6460 triple-quadrupole mass spectrometer (Agilent Technologies, Inc, USA). 1 μL of extract containing choline metabolites was injected into an analytical column, BEH Amide (Waters, USA, 2.1 × 100 mm, 1.7 μm). A binary gradient elution system of mobile phase A [ACN/water (50:50, v/v)] and mobile phase B [ACN/water (90:10, v/v)] was used, both A and B contained 10 mM ammonium formate and 0.1% formic acid. 0.5 mL/min flow rate and 40 °C column temperature was maintained. Linear elution was performed as follows: 100% B changed to 100% A from 0 to 5 min, and maintained for 2 min, then changed to 100%B and maintained for 3 min.

### RNA extraction and quantitative real-time PCR

Total mRNA was isolated using RNAiso plus (Takara Bio Inc., Japan). Complementary DNA was synthesized using PrimeScript^TM^ RT reagent Kit with gDNA Eraser (Takara Bio Inc.). Quantitative real-time PCR was performed by SYBR Premix Ex TagTMII (Takara Bio Inc.) and ABI Step1 (Applied Biosystems, Bedford, MA, USA). The primer sequences were obtained from the primer bank (https://pga.mgh.harvard.edu/primerbank/) and given in Table S5. Gene transcription data were normalized to β-actin.

### HBV DNA extraction and quantitative real-time PCR

HBV DNA was extracted from the cells based on the method of Sterneck *et al*.^41^. In brief, cells were washed in ice-cold PBS and lysed on ice for 10 min with 0.8 mL of lysis buffer containing 50 mM Tris-HCl, 50 mM NaCl, 1 mM EDTA, 1% NP-40 (pH 7.6). Nuclei in the supernatant was treated with 8 μL MgCl_2_ (1 M) and 8 μL DNase I (10 mg/mL) at 37 °C for 30 min. The reaction was stopped by the addition of 40 μL EDTA (0.5 M, pH 8.0). Proteins were then digested with 20 μL proteinase K (20 mg/mL) and 80 μL 10% sodium dodecyl sulfate at 55 °C for 2 h. The HBV DNA was purified by 500 uL Tris-phenol/chloroform (v/v = 1:1) and the upper extract was treated with 560 μL isopropanol, 15 μg tRNA and 80 μL sodium acetate (3 M, pH 5.2) at −20 °C overnight. The precipitation contained HBV DNA was washed with 1 mL 70% ethanol and re-dissolved in 20 μL waters and stored at −20 °C before analysis. The HBV DNA were quantified by real-time PCR using SYBR Premix Ex TagTMII (Takara Bio Inc.) and ABI Step1 (Applied Biosystems, Bedford, MA, USA). The primer sequences of HBV DNA were the same as described previously^11^.

### Cell transfection of small interfering RNA (siRNA)

The *PCYT1A* siRNA, *LPP1* siRNA and negative control siRNA used in the experiment were obtained from GenePharma (Shanghai, China). The siRNAs sequences were given in Table S6. HepG2.2.15 cells were transfected with siRNAs by using transfection reagent siRNA-mate (GenePharma) according to the manufacturer’s recommendation.

### Enzyme linked immunosorbent assay

The levels of HBV s antigen (HBsAg) and HBV e antigen (HBeAg) in the culture supernatants of cells were detected by kits (Kehua, Shanghai, China) according to the manufacturer’s instructions. The cell counts assay was performed before siRNA treatment (0 h), and at 6 h, 12 h, 24 h, 48 h, 72 h and 96 h after transfection using a cell counting kit-8 (CCK-8) (Dojindo, Japan).

### Western blot

Cells were homogenized in RIPA lysis buffer (Beyotime Biotechnology, Shanghai, China), and the protein concentrations of the lysates were determined by a BCA protein reagent kit (Beyotime Biotechnology). Anti-LPP1 antibody (or anti-PPAP2A antibody, Abcam, ab198280, 1:500), Anti-PCYT1A antibody (Abcam, ab109263, 1:1000) were used, and Anti-Actin antibody (Abcam, ab8226, 1:2000) was used to ensure equivalent loading of total protein.

### Statistical analysis

Univariate statistical analysis was performed on the normalized data using the Student’s t-test or Mann–Whitney U test, *p* value < 0.05 was considered to be statistically significant. In addition, multivariate statistical model was constructed using the orthogonal projection to latent structure-discriminant analysis (OPLS-DA) with mean-centering and scale to unit (UV) scaling and validated with a seven-fold cross validation method and permutation test using SIMCA-P+ (v12.0, Umetrics, Sweden). The significance of the OPLS-DA model was additionally validated by the analysis of variance in the cross-validated residuals (CV-ANOVA), *p* value < 0.05 indicated the validity of the model. The significance of the metabolites was selected with the criteria that satisfied the followings: variable importance in the projection (VIP) >1, *p* value < 0.05, and fold change >1.2 or < 0.8^42^. Classification models could also be characterized by receiver operating characteristic (ROC) curve, which is a probability curve, often plotted as true positive rate against false positive rate. ROC can be used to select the best diagnostic threshold. AUC is the area under the ROC curve. When AUC is greater than 0.5, or closer to 1, the better the diagnosis power would be.

## Supplementary information

Supplementary Information
