# Supplementary material for: Stimulated phospholipid synthesis is key for hepatitis B virus replications
Source: Sci Rep. 2019 Sep 10;9:12989. doi: 10.1038/s41598-019-49367-8 (PMC6736851; doi:10.1038/s41598-019-49367-8)
Supplement: Supplementary file 1 — Supplementary Information [file 41598_2019_49367_MOESM1_ESM.docx]

**Supplementary Information**

Stimulated phospholipid synthesis is key for hepatitis B virus replications

Qingxia Huang^1, 2^, Hehua Lei^1^, Laifeng Ding^1, 2^, Yulan Wang^3, *^

^1^State Key Laboratory of Magnetic Resonance and Atomic and Molecular Physics, National Center for Magnetic Resonance in Wuhan, Key Laboratory of Magnetic Resonance in Biological Systems, Wuhan Institute of Physics and Mathematics, Chinese Academy of Sciences, Wuhan 430071, P. R. China

^2^ University of Chinese Academy of Sciences, Beijing 100049, P. R. China

^3^ Singapore Phenome Center, Lee Kong Chian School of Medicine, Nanyang Technological University, Singapore

^*^ To whom the correspondences should be addressed: E-mail: [yulan.wang@ntu.edu.sg](mailto:yulan.wang@ntu.edu.sg), Fax: +65-65150417, Tel.: +65-69041106.


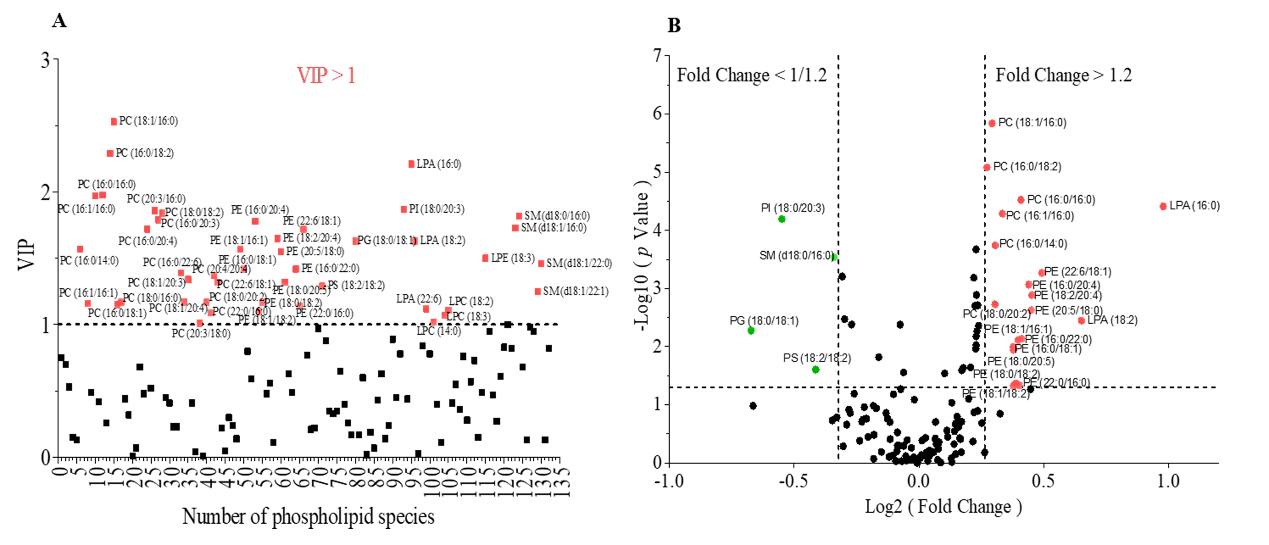


**Fig S1.** Identification of unique phospholipids. **A.** The variable importance in the projection (VIP) scores from PLS-DA model (VIP >1), high VIP score indicates great contribution to the separation between HBsAg (+) and HBsAg (-) group. Red, VIP > 1. **B.** Volcano plots of the significant phospholipids between HBsAg (+) and HBsAg (-) group. Phospholipids with a fold change threshold of > 1.2 or < 0.8 and t-tests *p* value < 0.05 are identified as significant. Green, *p* value < 0.05, and fold change < 0.8. Red, *p* value < 0.05, and fold change > 1.2. The significance of the phospholipids was used the criteria that VIP > 1, *p* value < 0.05, and fold change > 1.2 or < 0.8.


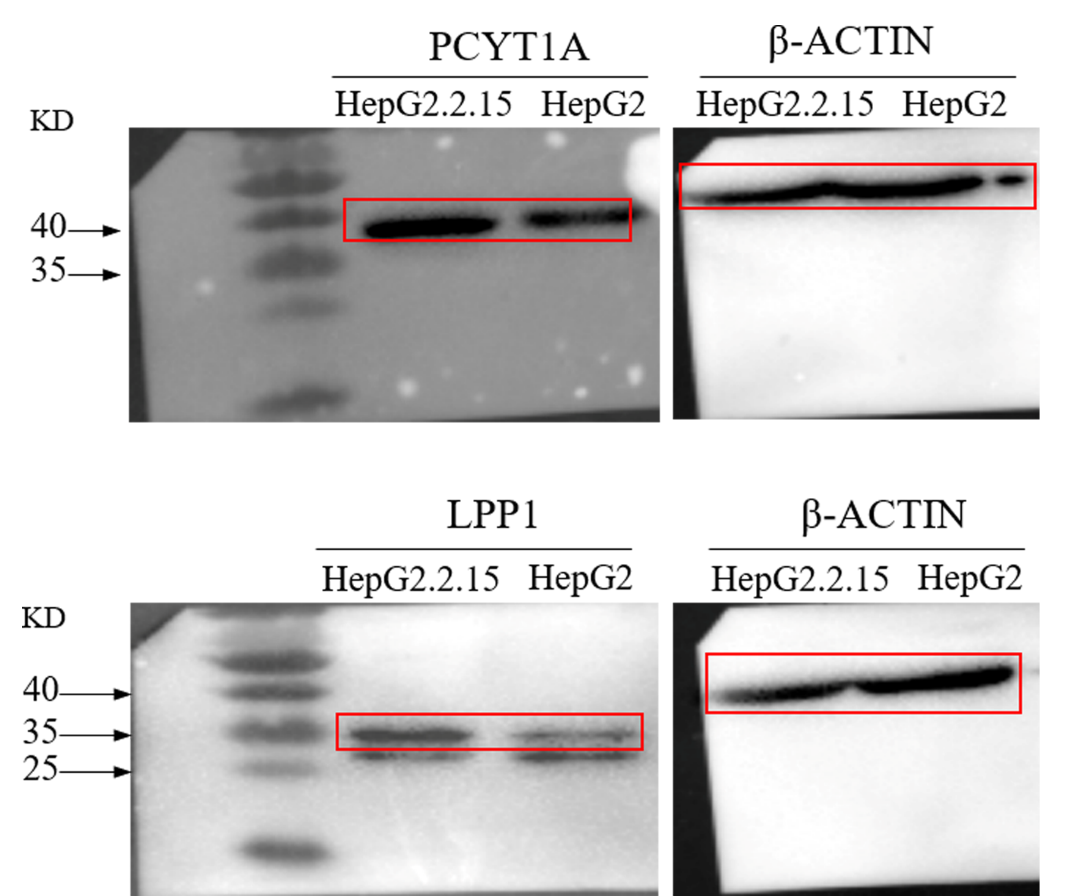


**Fig S2.** The western blot analysis of LPP1, PCYT1A protein expression levels in HepG2 and HepG2.2.15 cells.


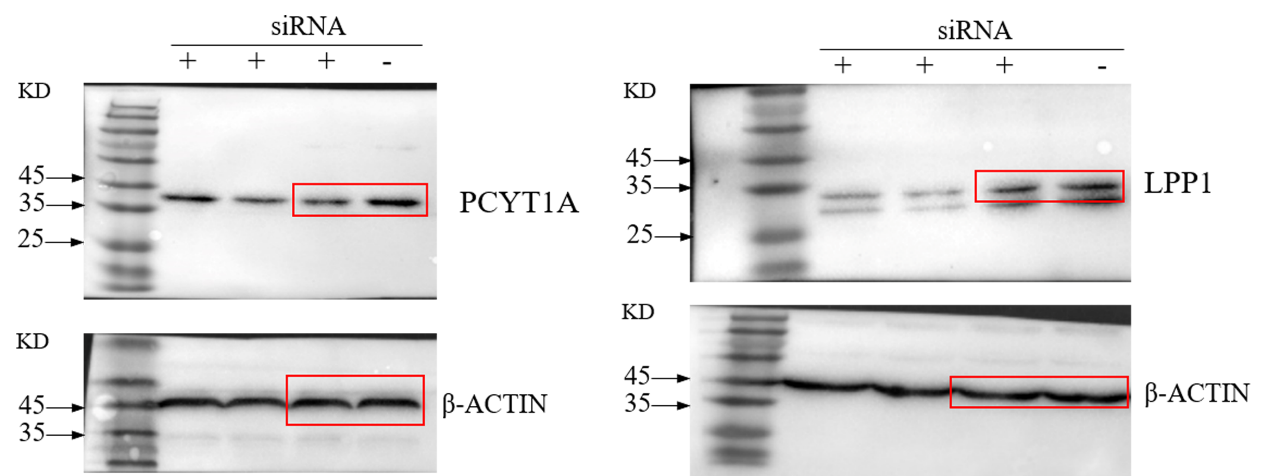


**Fig S3.** The western blot analysis of LPP1, PCYT1A protein expression levels in the siRNA negative control group and treatment group.

| **Table S1.** The concentrations of phospholipids extracted from the serum | | | | | | | |
| --- | --- | --- | --- | --- | --- | --- | --- |
| Phospholipids | HBsAg (-) | HBsAg (+) | p value ^a^ | Phospholipids | HBsAg (-) | HBsAg (+) | p value ^a^ |
| PC (14:1/14:1) | 0.06±0.03 | 0.07±0.03 | 0.078 | PE (20:1/20:4 | 0.46±0.29 | 0.48±0.28 | 0.569 |
| PC (12:0/16:1) | 1.20±0.44 | 1.33±0.48 | 0.159 | PE (20:3/20:1 | 0.14±0.09 | 0.15±0.09 | 0.436 |
| PC (14:1/16:1) | 3.86±1.85 | 4.23±1.48 | 0.092 | PS (16:0/18:0) | 4.29±2.68 | 3.37±1.60 | 0.185 |
| PC (14:1/16:0) | 22.96±7.30 | 23.38±5.92 | 0.370 | PS (18:2/18:2) | 1.64±0.86 | 1.23±0.58 | 0.025 |
| PC (14:0/16:0) | 1.66±0.70 | 1.70±0.66 | 0.657 | PS (18:0/18:1) | 0.65±0.42 | 0.52±0.27 | 0.164 |
| PC (16:0/14:0) | 1.18±0.43 | 1.46±0.38 | <0.001 | PS (18:0/18:0) | 0.23±0.12 | 0.22±0.07 | 0.927 |
| PC (16:1/16:1) | 2.30±0.96 | 2.71±0.65 | 0.002 | PA (18:0/18:2) | 9.01±4.86 | 8.31±5.46 | 0.197 |
| PC (16:0/16:1) | 4.27±2.67 | 4.81±2.64 | 0.194 | PA (18:0/22:6) | 10.42±4.58 | 9.81±3.95 | 0.627 |
| PC (16:1/16:0) | 3.49±1.06 | 4.39±0.98 | <0.001 | PG (16:1/18:2) | 9.16±2.56 | 9.90±2.91 | 0.276 |
| PC (14:0/18:0) | 0.48±0.32 | 0.54±0.32 | 0.241 | PG (16:0/20:5) | 1.39±0.42 | 1.46±0.37 | 0.197 |
| PC (16:0/16:0) | 11.57±3.72 | 15.35±4.81 | <0.001 | PG (18:2/18:2) | 1.78±0.67 | 1.86±0.71 | 0.651 |
| PC (20:3/14:1) | 0.86±0.48 | 0.81±0.45 | 0.503 | PG (18:1/18:1) | 0.13±0.05 | 0.13±0.05 | 0.927 |
| PC (16:0/18:2) | 143.45±23.95 | 173.16±31.67 | <0.001 | PG (18:0/18:1) | 0.09±0.06 | 0.06±0.03 | 0.005 |
| PC (18:1/16:0) | 11.30±1.70 | 13.84±2.45 | <0.001 | PI (16:0/16:0) | 0.21±0.12 | 0.22±0.12 | 0.651 |
| PC (16:0/18:1) | 82.35±20.96 | 92.74±21.79 | 0.025^b^ | PI (16:0/18:2) | 3.09±1.32 | 3.46±1.63 | 0.374 |
| PC (18:0/16:0) | 10.95±2.84 | 12.38±2.96 | 0.023^b^ | PI (18:1/16:0) | 2.16±0.92 | 2.15±0.88 | 0.993 |
| PC (16:0/18:0) | 4.60±2.09 | 4.98±2.10 | 0.357 | PI (16:0/18:0) | 0.15±0.07 | 0.14±0.06 | 0.907 |
| PC (14:1/22:6) | 0.32±0.13 | 0.30±0.09 | 0.933 | PI (16:0/20:4) | 2.85±1.37 | 2.89±1.28 | 0.744 |
| PC (16:1/20:5) | 0.38±0.23 | 0.38±0.20 | 0.814 | PI (18:1/18:2) | 1.53±0.68 | 1.68±0.97 | 0.960 |
| PC (18:3/18:2) | 0.94±0.56 | 0.96±0.67 | 0.712 | PI (18:1/18:1) | 2.12±1.25 | 1.82±1.05 | 0.180 |
| PC (16:0/20:5) | 8.92±4.76 | 7.74±3.27 | 0.357 | PI (18:0/18:2) | 9.37±3.44 | 9.18±3.58 | 0.546 |
| PC (18:2/18:2) | 15.78±7.09 | 17.47±10.12 | 0.700 | PI (18:1/18:0) | 0.35±0.15 | 0.33±0.15 | 0.508 |
| PC (16:0/20:4) | 62.07±11.93 | 72.25±15.06 | 0.001 | PI (18:0/18:1) | 2.15±1.14 | 1.77±0.86 | 0.121 |
| PC (18:1/18:2) | 1.47±0.70 | 1.65±1.00 | 0.651 | PI (18:1/20:4) | 1.06±0.41 | 1.16±0.66 | 0.828 |
| PC (20:3/16:0) | 4.85±0.88 | 5.68±1.13 | <0.001 | PI (18:0/20:4) | 29.77±12.55 | 26.28±8.06 | 0.327 |
| PC (16:0/20:3) | 71.41±11.42 | 83.40±18.62 | 0.002 | PI (18:0/20:3) | 2.51±1.03 | 1.71±0.84 | <0.001 |
| PC (18:0/18:2) | 6.52±1.04 | 7.65±1.69 | 0.001 | PI (18:0/22:6) | 0.91±0.41 | 0.84±0.27 | 0.737 |
| PC (18:2/18:0) | 132.99±37.47 | 140.09±39.85 | 0.503 | LPA (16:0) | 2.21±1.45 | 4.35±2.68 | <0.001 |
| PC (18:1/18:0) | 13.13±3.77 | 13.78±3.92 | 0.476 | LPA (18:2) | 2.09±1.38 | 3.28±1.98 | 0.004 |
| PC (18:0/18:1) | 30.85±15.66 | 32.28±14.51 | 0.393 | LPA (20:4) | 2.02±1.28 | 2.01±1.14 | 0.953 |
| PC (18:0/18:0) | 4.53±2.34 | 4.74±2.16 | 0.379 | LPA (20:1) | 3.52±2.25 | 4.41±2.86 | 0.143 |
| PC (16:0/22:6) | 41.49±12.35 | 48.73±11.83 | 0.005 | LPA (22:6) | 5.40±3.46 | 7.35±4.85 | 0.054 |
| PC (18:1/20:4) | 23.05±6.97 | 26.59±7.29 | 0.023^b^ | LPA (22:1) | 2.51±1.37 | 2.99±1.59 | 0.206 |
| PC (18:1/20:3) | 9.57±2.42 | 11.21±3.31 | 0.009 | LPC (14:0) | 0.47±0.26 | 0.38±0.13 | 0.513 |
| PC (18:0/20:4) | 43.13±15.33 | 40.79±11.67 | 0.503 | LPC (16:1) | 1.44±0.49 | 1.37±0.38 | 0.004 |
| PC (18:0/20:3) | 33.23±15.82 | 33.48±14.05 | 0.731 | LPC (16:0) | 117.61±35.38 | 116.12±29.92 | 0.081 |
| PC (20:3/18:0) | 12.93±5.44 | 15.13±4.93 | 0.011 | LPC (18:3) | 0.50±0.21 | 0.41±0.17 | 0.138 |
| PC (20:2/18:0) | 3.52±1.72 | 3.52±1.51 | 0.828 | LPC (18:2) | 24.38±7.88 | 20.68±7.84 | 0.416 |
| PC (18:0/20:2) | 22.93±11.45 | 28.30±10.04 | 0.002 | LPC (18:1) | 22.98±5.73 | 22.02±5.48 | 0.028 |
| PC (22:0/16:0) | 1.34±0.56 | 1.57±0.41 | 0.007 | LPC (18:0) | 59.76±23.84 | 54.76±19.05 | 0.172 |
| PC (20:4/20:4) | 0.76±0.25 | 0.61±0.23 | 0.003 | LPC (20:5) | 0.32±0.17 | 0.29±0.17 | 0.782 |
| PC (22:6/18:1) | 2.18±0.69 | 2.57±0.70 | 0.004 | LPC (20:4) | 5.28±1.98 | 4.66±1.95 | 0.847 |
| PC (22:6/18:0) | 12.50±4.64 | 12.86±3.24 | 0.609 | LPC (20:3) | 1.80±0.58 | 1.72±0.97 | 0.054 |
| PC (20:1/20:4) | 4.73±2.43 | 4.77±1.50 | 0.412 | LPC (20:2) | 1.05±0.33 | 1.13±0.33 | 0.029 |
| PC (20:0/20:5) | 3.14±1.35 | 3.33±1.85 | 0.980 | LPC (20:1) | 1.05±0.50 | 1.22±0.63 | 0.427^b^ |
| PC (20:3/20:1) | 1.72±0.83 | 1.81±1.01 | 0.880 | LPC (22:6) | 1.28±0.48 | 1.24±0.84 | 0.402 |
| PE (14:0/20:3) | 0.17±0.11 | 0.17±0.11 | 0.763 | LPE (16:0) | 1.83±0.73 | 1.69±0.67 | 0.388 |
| PE (18:1/16:1) | 1.01±0.38 | 1.34±0.59 | 0.007 | LPE (18:3) | 0.13±0.08 | 0.08±0.05 | 0.104 |
| PE (16:0/18:1) | 0.09±0.04 | 0.12±0.05 | 0.010 | LPE (18:2) | 3.88±1.83 | 3.17±1.67 | 0.218 |
| PE (18:1/16:0) | 0.79±0.37 | 0.92±0.47 | 0.127 | LPE (18:1) | 3.39±1.34 | 3.09±1.70 | 0.138 |
| PE (20:4/16:0) | 0.66±0.44 | 0.79±0.64 | 0.657 | LPE (18:0) | 5.16±2.56 | 4.86±2.87 | 0.299 |
| PE (16:0/20:4) | 1.46±0.52 | 1.98±0.80 | 0.001 | LPE (20:4) | 1.21±0.47 | 1.06±0.66 | 0.104 |
| PE (18:1/18:2) | 2.44±1.05 | 3.17±2.02 | 0.047 | SM (d18:1-14:1) | 0.23±0.11 | 0.19±0.08 | 0.194 |
| PE (18:0/18:2) | 0.22±0.10 | 0.29±0.18 | 0.046 | SM (d18:1-14:0) | 3.47±1.24 | 2.99±1.07 | 0.109 |
| PE (18:1/18:1) | 7.56±3.55 | 8.33±4.21 | 0.471 | SM (d18:1-16:1) | 13.23±5.74 | 11.44±4.58 | 0.172 |
| PE (18:1/18:0) | 0.44±0.21 | 0.49±0.26 | 0.388 | SM (d18:1-16:0) | 76.47±19.89 | 61.81±18.51 | 0.001 |
| PE (18:0/18:1) | 1.65±1.10 | 1.70±1.04 | 0.750 | SM (d18:0-16:0) | 5.21±1.39 | 4.11±1.31 | <0.001 |
| PE (18:2/20:4) | 3.75±1.56 | 5.13±2.26 | 0.001 | SM (d18:1-18:1) | 6.75±2.83 | 6.09±1.59 | 0.645 |
| PE (20:5/18:0) | 1.11±0.51 | 1.51±0.69 | 0.002 | SM (d18:1-18:0) | 9.89±3.01 | 9.75±1.99 | 0.789 |
| PE (18:0/20:5) | 0.69±0.32 | 0.89±0.41 | 0.011 | SM (d18:1-20:1) | 4.32±1.34 | 3.84±0.92 | 0.115 |
| PE (20:4/18:0) | 4.49±1.78 | 4.96±1.85 | 0.212 | SM (d18:1-20:0) | 62.27±10.67 | 58.00±10.76 | 0.066^b^ |
| PE (18:0/20:3) | 1.18±0.61 | 1.30±0.59 | 0.283 | SM (d18:1-22:1) | 30.41±6.03 | 27.21±6.05 | 0.015^b^ |
| PE (16:0/22:0) | 0.14±0.06 | 0.18±0.08 | 0.008 | SM (d18:1-22:0) | 58.67±16.68 | 48.65±14.90 | 0.004 |
| PE (22:0/16:0) | 0.13±0.07 | 0.17±0.10 | 0.043 | SM (d18:1-24:1) | 54.59±25.57 | 53.35±18.53 | 0.867 |
| PE (22:6/18:1) | 0.30±0.13 | 0.42±0.19 | 0.001 | SM (d18:0-24:1) | 14.59±7.00 | 12.19±6.97 | 0.064 |
| PE (18:0/22:6) | 1.83±0.95 | 2.13±0.93 | 0.134 |  |  |  |  |
| Data are shown as mean ± SD, nmol/mL. n [HBsAg (-)] = 48, n [HBsAg (+)] = 40. ^a^: Mann-Whitney U test, ^b^: t-test. Key: PC: phosphatidylcholine, PE: phosphatidylethanolamine, PS: phosphatidylserine, PA: phosphatidic acid, PG: phosphatidylglycerol, PI: phosphatidylinositol, LPA: lyso-phosphatidic acid, LPC: lyso-phosphatidylcholine, LPE: lyso-phosphatidylethanolamine, SM: sphingomyelin. Details of the phospholipid composition of each sample were shown in “Supplementary dataset 1”. | | | | | | | |

| **Table S2.** ROC analysis of serum and the AUC Results | | | | |
| --- | --- | --- | --- | --- |
| Phospholipids | AUC |  | Phospholipids | AUC |
| PC (16:0/14:0) | 0.733 |  | PE (20:5/18:0) | 0.689 |
| PC (16:1/16:0) | 0.752 |  | PE (18:0/20:5) | 0.657 |
| PC (16:0/16:0) | 0.759 |  | PE (16:0/22:0) | 0.666 |
| PC (16:0/18:2) | 0.777 |  | PE (22:0/16:0) | 0.626 |
| PC (18:1/16:0) | 0.799 |  | PE (22:6/18:1) | 0.715 |
| PC (18:0/20:2) | 0.693 |  | PS (18:2/18:2) | 0.360 |
| PE (18:1/16:1) | 0.667 |  | PG (18:0/18:1) | 0.327 |
| PE (16:0/18:1) | 0.660 |  | PI (18:0/20:3) | 0.252 |
| PE (16:0/20:4) | 0.707 |  | LPA (16:0) | 0.756 |
| PE (18:1/18:2) | 0.623 |  | LPA (18:2) | 0.681 |
| PE (18:0/18:2) | 0.624 |  | SM (d18:0/16:0 | 0.283 |
| PE (18:2/20:4) | 0.700 |  |  |  |
| ROC: Receiver-operating characteristic  AUC: Area under the receiver-operating characteristic | | | | |

| **Table S3.** The concentrations of phospholipids species extracted from the cells | | | | | | | |
| --- | --- | --- | --- | --- | --- | --- | --- |
| Phospholipids | HepG2 | HepG2.2.15 | *p* value | Phospholipids | HepG2 | HepG2.2.15 | *p* value |
| PC (14:0/14:1) | 3.41+0.70 | 2.82+0.88 | 0.134 | PA (16:0/16:0) | 0.52+0.23 | 0.22+0.04 | 0.001 |
| PC (14:0/14:0) | 3.44+0.84 | 2.42+0.59 | 0.009 | PA (16:0/18:2) | 0.18+0.05 | 0.27+0.10 | 0.035 |
| PC (18:2/12:0) | 1.82+0.26 | 4.22+1.23 | <0.001 | PA (16:0/18:1) | 2.85+0.65 | 2.87+1.06 | 0.976 |
| PC (14:0/16:1) | 9.70+2.20 | 9.37+2.21 | 0.750 | PA (18:1/18:1) | 4.29+1.33 | 2.18+0.68 | 0.001 |
| PC (18:1/12:0) | 11.82+3.07 | 21.09+4.56 | 0.001 | PA (18:0/18:1) | 6.23+1.65 | 6.61+2.68 | 0.728 |
| PC (14:0/16:0) | 0.87+0.18 | 0.88+0.23 | 0.895 | PA (18:0/20:2) | 0.37+0.08 | 0.23+0.07 | 0.001 |
| PC (16:0/14:0) | 10.33+4.53 | 14.46+2.81 | 0.009 | PA (18:0/22:6) | 0.29+0.08 | 1.03+0.49 | 0.001 |
| PC (16:1/16:1) | 15.98+3.36 | 12.67+2.52 | 0.047 | PG (16:0/16:1) | 5.92+1.34 | 1.48+0.41 | 0.000 |
| PC (16:0/16:1) | 33.78+13.25 | 46.76+10.18 | 0.012 | PG (16:1/16:0) | 0.93+0.39 | 0.61+0.22 | 0.051 |
| PC (16:1/16:0) | 3.22+0.87 | 3.54+0.71 | 0.171 | PG (16:1/18:2) | 6.37+1.75 | 0.72+0.34 | <0.001 |
| PC (14:0/18:0) | 3.50+1.61 | 5.06+1.13 | 0.009 | PG (16:1/18:1) | 74.65+17.18 | 11.14+3.66 | <0.001 |
| PC (16:0/16:0) | 10.21+4.07 | 28.74+6.63 | <0.001 | PG (16:0/18:1) | 16.01+3.50 | 10.12+2.12 | 0.001 |
| PC (14:0/20:4) | 1.36+0.18 | 2.14+0.53 | 0.002 | PG (18:1/18:2) | 28.78+7.00 | 4.61+1.53 | <0.001 |
| PC (16:1/18:2) | 2.70+0.84 | 3.59+0.65 | 0.009 | PG (18:1/18:1) | 159.92+41.66 | 41.71+14.02 | <0.001 |
| PC (16:0/18:3) | 3.28+0.61 | 2.88+0.48 | 0.137 | PG (18:0/18:1) | 5.42+1.64 | 8.88+3.92 | 0.058 |
| PC (16:0/18:2) | 26.51+10.22 | 37.08+8.63 | 0.009 | PG (18:0/18:0) | 0.25+0.08 | 0.68+0.27 | 0.005 |
| PC (18:1/16:0) | 2.12+0.87 | 3.13+0.81 | 0.012 | PG (18:1/20:4) | 9.17+2.26 | 1.76+0.57 | <0.001 |
| PC (16:0/18:1) | 32.46+14.39 | 63.18+17.42 | 0.002 | PG (18:1/20:3) | 38.15+8.45 | 22.24+7.99 | 0.001 |
| PC (18:0/16:0) | 3.92+1.68 | 7.68+2.11 | 0.002 | PI (16:0/16:1) | 0.29+0.07 | 0.17+0.06 | 0.001 |
| PC (16:0/18:0) | 3.04+0.55 | 19.15+4.18 | <0.001 | PI (16:0/16:0) | 0.29+0.10 | 0.11+0.03 | <0.001 |
| PC (16:0/20:5) | 1.98+0.34 | 8.00+1.95 | <0.001 | PI (16:1/18:1) | 5.04+1.70 | 1.26+0.40 | <0.001 |
| PC (18:2/18:2) | 0.77+0.24 | 1.90+0.45 | <0.001 | PI (18:1/16:0) | 5.90+1.56 | 2.13+0.55 | <0.001 |
| PC (16:0/20:4) | 3.83+1.13 | 16.42+4.79 | <0.001 | PI (16:1/18:0) | 0.36+0.16 | 0.15+0.05 | 0.001 |
| PC (20:3/16:0) | 10.95+3.13 | 9.47+2.72 | 0.310 | PI (16:0/18:0) | 0.53+0.25 | 0.16+0.05 | <0.001 |
| PC (16:0/20:3) | 9.03+1.74 | 22.25+4.07 | <0.001 | PI (16:0/20:4) | 8.37+1.73 | 8.75+3.31 | 0.762 |
| PC (16:0/20:2) | 1.68+0.35 | 2.77+0.55 | 0.001 | PI (18:1/18:1) | 44.84+13.43 | 21.26+7.75 | <0.001 |
| PC (18:1/18:1) | 45.31+10.58 | 86.23+15.84 | <0.001 | PI (18:0/18:2) | 18.29+6.75 | 3.32+1.08 | <0.001 |
| PC (18:1/18:0) | 4.80+1.04 | 8.30+1.50 | 0.001 | PI (18:0/18:1) | 5.91+1.63 | 3.38+1.38 | 0.003 |
| PC (18:0/18:1) | 29.35+4.14 | 72.41+14.68 | <0.001 | PI (18:0/18:0) | 0.48+0.13 | 0.32+0.13 | 0.015 |
| PC (18:0/18:0) | 3.99+0.56 | 9.69+1.99 | <0.001 | PI (18:1/20:4) | 23.98+6.26 | 26.82+10.10 | 0.484 |
| PC (16:1/22:6) | 0.24+0.03 | 0.57+0.16 | <0.001 | PI (18:0/20:4) | 83.88+21.78 | 165.90+65.75 | 0.007 |
| PC (16:0/22:6) | 0.68+0.22 | 4.22+1.13 | <0.001 | PI (20:3/18:0) | 7.17+1.76 | 10.39+3.73 | 0.038 |
| PC (18:1/20:4) | 0.35+0.09 | 1.37+0.24 | <0.001 | PI (18:0/20:3) | 270.83+83.52 | 33.36+14.39 | <0.001 |
| PC (18:0/20:5) | 3.24+0.96 | 13.19+3.50 | <0.001 | PI (20:2/18:0) | 22.53+6.94 | 9.08+3.47 | <0.001 |
| PC (20:2/18:1) | 0.29+0.11 | 1.21+0.31 | <0.001 | LPA (16:0) | 1.89+1.21 | 1.08+0.30 | 0.122 |
| PC (18:1/20:3) | 2.03+0.59 | 8.04+1.96 | <0.001 | LPA (18:3) | 11.55+3.77 | 7.97+2.04 | 0.023 |
| PC (18:0/20:4) | 4.38+0.74 | 9.86+1.95 | <0.001 | LPA (18:2) | 5.05+1.34 | 2.53+0.95 | <0.001 |
| PC (18:0/20:3) | 11.42+1.89 | 21.13+3.66 | <0.001 | LPA (18:1) | 3.37+1.17 | 2.14+0.48 | 0.009 |
| PC (20:3/18:0) | 4.95+0.86 | 3.77+0.67 | 0.005 | LPA (20:4) | 6.88+1.86 | 3.51+1.14 | <0.001 |
| PC (20:1/18:1) | 10.86+2.82 | 24.97+6.97 | <0.001 | LPA (20:1) | 56.47+17.92 | 38.34+12.19 | 0.023 |
| PC (18:1/20:1) | 8.00+2.39 | 14.65+5.71 | 0.007 | LPA (20:0) | 5.10+2.67 | 3.44+0.91 | 0.200 |
| PE (16:1/16:1) | 19.97+4.18 | 3.01+1.56 | <0.001 | LPA (22:6) | 7.89+2.91 | 8.53+2.67 | 0.632 |
| PE (16:0/16:1) | 25.06+5.06 | 9.38+1.68 | <0.001 | LPA (22:2) | 10.81+3.11 | 7.74+1.73 | 0.020 |
| PE (16:0/16:0) | 1.56+0.39 | 1.11+0.23 | 0.007 | LPA (22:1) | 7.81+2.57 | 5.57+1.53 | 0.039 |
| PE (16:0/18:2) | 57.79+14.25 | 16.69+2.72 | <0.001 | LPA (24:1) | 8.93+2.73 | 6.76+2.33 | 0.089 |
| PE (16:0/18:1) | 6.33+1.62 | 1.80+0.27 | <0.001 | LPC (14:0) | 0.96+0.15 | 0.32+0.05 | <0.001 |
| PE (18:1/16:0) | 32.20+7.86 | 24.44+4.33 | 0.005 | LPC (16:1) | 3.94+0.57 | 1.76+0.21 | <0.001 |
| PE (16:0/18:0) | 2.10+0.46 | 1.65+0.31 | 0.015 | LPC (16:0) | 44.10+4.97 | 29.95+6.67 | <0.001 |
| PE (18:0/16:0) | 3.17+0.81 | 6.57+2.56 | 0.002 | LPC (18:2) | 1.12+0.15 | 1.54+0.32 | 0.003 |
| PE (20:4/16:1) | 14.59+2.51 | 13.31+4.88 | 0.757 | LPC (18:1) | 26.77+2.34 | 23.35+5.71 | 0.070 |
| PE (20:4/16:0) | 1.31+0.21 | 1.15+0.46 | 0.825 | LPC (18:0) | 17.02+1.38 | 23.33+5.46 | 0.015 |
| PE (16:0/20:4) | 39.97+6.63 | 31.02+5.79 | 0.008 | LPC (20:4) | 0.35+0.13 | 1.34+0.35 | <0.001 |
| PE (18:3/18:0) | 3.32+0.53 | 2.65+0.48 | 0.013 | LPC (20:3) | 1.23+0.19 | 1.02+0.18 | 0.028 |
| PE (18:2/18:1) | 39.57+18.05 | 21.69+4.67 | <0.001 | LPC (20:2) | 0.92+0.10 | 0.59+0.13 | <0.001 |
| PE (18:1/18:2) | 82.34+11.22 | 17.62+3.20 | <0.001 | LPC (20:1) | 1.80+0.26 | 2.57+0.71 | 0.012 |
| PE (18:0/18:2) | 11.12+1.73 | 3.43+0.61 | <0.001 | LPC (20:0) | 0.25+0.04 | 0.42+0.12 | 0.003 |
| PE (18:1/18:1) | 207.85+35.17 | 144.73+34.63 | 0.001 | LPE (14:1) | 0.25+0.16 | 0.32+0.17 | 0.453 |
| PE (18:1/18:0) | 20.52+3.63 | 12.83+2.68 | <0.001 | LPE (16:1) | 1.63+0.31 | 0.32+0.17 | <0.001 |
| PE (18:0/18:1) | 97.57+24.78 | 139.30+52.73 | 0.145 | LPE (16:0) | 30.85+5.82 | 9.62+3.07 | <0.001 |
| PE (18:0/18:0) | 7.00+1.84 | 9.85+3.72 | 0.145 | LPE (18:2) | 1.61+0.30 | 0.62+0.14 | <0.001 |
| PE (16:0/22:6) | 0.27+0.04 | 0.11+0.04 | <0.001 | LPE (18:1) | 155.89+27.56 | 54.61+19.12 | <0.001 |
| PE (18:1/20:5) | 11.35+1.84 | 18.65+5.36 | 0.009 | LPE (18:0) | 76.40+12.61 | 68.99+28.56 | 0.310 |
| PE (18:1/20:4) | 108.79+17.77 | 123.44+26.65 | 0.189 | LPE (20:4) | 0.95+0.22 | 1.70+0.42 | <0.001 |
| PE (18:1/20:3) | 9.90+1.93 | 10.85+2.34 | 0.360 | LPE (20:3) | 5.23+1.29 | 0.98+0.47 | <0.001 |
| PE (20:4/18:0) | 47.61+7.65 | 35.47+7.79 | 0.004 | LPE (20:2) | 3.60+0.87 | 0.93+0.31 | <0.001 |
| PE (18:0/20:4) | 178.62+22.83 | 173.37+38.35 | 0.729 | LPE (20:1) | 100.41+24.79 | 105.54+15.82 | 0.310 |
| PE (20:2/18:1) | 14.82+2.12 | 14.09+2.73 | 0.453 | SM (d18:1-14:1) | 1.51+0.91 | 1.07+0.76 | 0.233 |
| PE (20:3/18:0) | 122.26+34.77 | 92.10+33.41 | 0.079 | SM (d18:1-14:0) | 64.51+21.82 | 39.75+20.42 | 0.024 |
| PE (18:0/20:3) | 293.68+82.83 | 61.29+28.23 | <0.001 | SM (d18:1-16:1) | 34.49+10.10 | 62.77+31.14 | 0.058 |
| PE (18:1/20:1) | 33.88+12.80 | 26.88+11.12 | 0.233 | SM (d18:1-16:0) | 228.30+31.99 | 298.30+77.32 | 0.047 |
| PE (18:1/22:6) | 10.38+1.56 | 8.97+1.80 | 0.094 | SM (d18:1-18:1) | 4.02+0.53 | 6.04+1.57 | 0.005 |
| PE (20:0/20:5) | 28.16+6.58 | 27.34+8.30 | 0.965 | SM (d18:1-18:0) | 27.83+3.47 | 24.52+4.48 | 0.099 |
| PS (16:0/18:1) | 24.77+8.27 | 32.86+11.32 | 0.145 | SM (d18:0-20:6) | 0.42+0.07 | 0.34+0.07 | 0.020 |
| PS (18:0/16:1) | 13.36+4.72 | 12.30+4.18 | 0.965 | SM (d18:0-20:5) | 0.22+0.04 | 0.71+0.33 | <0.001 |
| PS (16:0/18:0) | 11.88+3.73 | 5.72+2.50 | <0.001 | SM (d18:1-20:2) | 3.79+0.84 | 4.45+2.01 | 0.691 |
| PS (18:2/18:2) | 2.35+0.68 | 0.71+0.17 | <0.001 | SM (d18:1-20:0) | 70.23+16.05 | 70.69+13.81 | 0.757 |
| PS (18:1/18:1) | 18.46+6.98 | 30.67+11.66 | 0.047 | SM (d18:0-20:0) | 85.61+22.21 | 121.58+24.54 | 0.019 |
| PS (18:0/18:1) | 28.31+10.86 | 60.35+27.69 | 0.012 | SM (d18:1-22:6) | 15.21+3.47 | 18.14+6.50 | 0.251 |
| PS (18:0/18:0) | 6.07+1.41 | 8.83+3.99 | 0.270 | SM (d18:1-22:1) | 52.89+6.78 | 58.95+12.53 | 0.220 |
| PS (18:0/20:4) | 40.83+15.18 | 29.19+9.62 | 0.122 | SM (d18:1-22:0) | 119.25+21.52 | 165.73+42.27 | 0.010 |
| PS (18:1/20:1) | 0.95+0.31 | 1.65+0.51 | 0.003 | SM (d18:1-24:1) | 151.22+65.82 | 202.09+119.33 | 0.279 |
| Data are shown as mean ± SD, nmol/g. n = 10, t-test. Key: PC: phosphatidylcholine, PE: phosphatidylethanolamine, PS: phosphatidylserine, PA: phosphatidic acid, PG: phosphatidylglycerol, PI: phosphatidylinositol, LPA: lyso-phosphatidic acid, LPC: lyso-phosphatidylcholine, LPE: lyso-phosphatidylethanolamine, SM: sphingomyelin. Details of the phospholipid composition of each sample were shown in “Supplementary dataset 2”. | | | | | | | |

| **Table S4.** The relative mRNA levels in HepG2 and HepG2.2.15 cells. | | | |
| --- | --- | --- | --- |
| Gene | HepG2 | HepG2.2.15 | *p* value |
| *LPP1* | 1.015±0.003 | 2.518±0.719 | 0.022 |
| *LPP3* | 1.004±0.001 | 0.958±0.258 | 0.775 |
| *LPIN1* | 1.002±0.002 | 0.935±0.067 | 0.159 |
| *LPIN2* | 1.003±0.002 | 0.505±0.035 | <0.001 |
| *LPIN3* | 1.002±0.001 | 0.651±0.014 | <0.001 |
| *DGKA* | 1.006±0.005 | 0.408±0.028 | <0.001 |
| *DGKE* | 1.006±0.006 | 0.244±0.040 | <0.001 |
| *DGKZ* | 1.014±0.017 | 0.673±0.035 | 0.001 |
| *PLA2G4A* | 1.013±0.014 | 1.016±0.109 | 0.967 |
| *PLA2G5* | 1.036±0.038 | 0.905±0.408 | 0.608 |
| *CHKA* | 1.005±0.003 | 1.017±0.200 | 0.923 |
| *CHKB* | 1.003±0.005 | 1.022±0.241 | 0.897 |
| *PCYT1A* | 1.010±0.006 | 1.665±0.296 | 0.019 |
| *PCYT1B* | 1.028±0.029 | 1.056±0.284 | 0.871 |
| *PEMT* | 1.016±0.019 | 0.679±0.077 | 0.002 |
| *PTDSS1* | 1.020±0.008 | 0.628±0.032 | 0.001 |
| *DGAT1* | 1.012±0.009 | 1.043±0.314 | 0.870 |
| *DGAT2* | 1.024±0.017 | 0.989±0.369 | 0.878 |
| Data are shown as mean ±SD, n=3, t-test. *LPP1*: lipid phosphate phosphatase 1, *LPP3*: lipid phosphate phosphatase 3, *LPIN1*: lipid phosphate phosphatase LPIN1, *LPIN2*: phosphatidate phosphatase LPIN2, *LPIN3*: phosphatidate phosphatase LPIN3, *DGKA*: diacylglycerol kinase A, *DGKZ*: diacylglycerol kinase Z, *DGKE*: diacylglycerol kinase E, *CHKA*: choline kinase A, *CHKB*: choline kinase B, *PCYT1A*: choline-phosphate cytidylyltransferase 1 A, *PCYT1B*: choline-phosphate cytidylyltransferase 1 B, *PEMT*: phosphatidylethanolamine N-methyltransferase, *PTDSS1*: phosphatidylserine synthase 1, *PLA2G4A*: cytosolic phospholipase A2 group Ⅳ A, *PLA2G5*: cytosolic phospholipase A2 group Ⅴ | | | |

| **Table S5.** The Primer sequences | | |
| --- | --- | --- |
| Gene | Forward Primer | Reverse Primer |
| *LPP1* | ACGCCCCACACTGCAATTT | TGAGTCCAGTCAACACATCGC |
| *LPP2* | TCTATTCTCGCTCGGACTTCA | TCAGACGCCCAATCATGTACT |
| *LPP3* | CTACGTGGCAGCACTCTATAAG | CCCTATGGACACTTTGGCAAT |
| *LPIN1* | CAGAGTTGTTGCCTCCGTTGT | AGAAATCTGCGATCGATGGC |
| *LPIN2* | GGATCACAGGAGCTCGAAGAA | TTTGCGATCTGGTCTGAGGAG |
| *LPIN3* | GGAGGAAACCCAAGCAGAAAG | AGGGATAGCTCACTCTCAGCG |
| *DGKA* | AATACCTGGATTGGGATGTGTCT | GTCCGTCGTCCTTCAGAGTC |
| *DGKZ* | CGGAGGCCCCAGAATACTCT | TTGTCGGGGATTGAGATACCA |
| *DGKE* | CTGGGAACAGGCAACGATCTA | CCATTACATTTCGCAAAACCTGC |
| *CHKA* | ATTACAGGGGATTCGACATTGGA | GCTGTTGTTTCTTGGTGGGAT |
| *CHKB* | CGGCTGGAACAGTACATCCC | GCCATGAAATTGCGCCATCT |
| *PCYT1A* | TCACGGTGATGAACGAGAATG | CCTCTGTGTTGGAGCAAACAT |
| *PCYT1B* | CCATGAAAAACTGACCATTGCTC | GCATAAGGGCTCTTGCATGAC |
| *PEMT* | GGGGTTCGCTGGAACTTTC | GAGCCACTATGTAGGTGAGGG |
| *PTDSS1* | GCAAGTGGAGGACATCACCAT | TCATCCCTGGTAAAGGCGAAG |
| *PLA2G4A* | AATACTGCACAATGCCCTTTACC | GCTTCCAAATAAGTCGGGAGC |
| *PLA2G5* | AAGGAGGCTTGCTGGACCTAA | GCGAATGTTGCAGCCCTTC |
| *β-actin* | TGTGTTGGCGTACAGGTCTTTG | GGGAAATCGTGCGTGACATTAAG |
| *LPP1*: lipid phosphate phosphatase 1, *LPP2*: lipid phosphate phosphatase 2, *LPP3*: lipid phosphate phosphatase 3, *LPIN1*: phosphatidate phosphatase LPIN1, *LPIN2*: phosphatidate phosphatase LPIN2, *LPIN3*: phosphatidate phosphatase LPIN3, *DGKA*: diacylglycerol kinase A, *DGKZ*: diacylglycerol kinase Z, *DGKE*: diacylglycerol kinase E, *CHKA*: choline kinase A, *CHKB*: choline kinase B, *PCYT1A*: choline-phosphate cytidylyltransferase 1 A, *PCYT1B*: choline-phosphate cytidylyltransferase 1 B, *PEMT*: phosphatidylethanolamine N-methyltransferase, *PTDSS1*: phosphatidylserine synthase 1, *PLA2G4A*: cytosolic phospholipase A2 group Ⅳ A, *PLA2G5*: cytosolic phospholipase A2 group Ⅴ.  The primer sequences were obtained from the primer bank (https://pga.mgh.harvard.edu/primerbank/.) | | |

| **Table S6. The siRNA sequences** | | |
| --- | --- | --- |
| Gene | siRNA sequences | |
|  | sense（5'-3'） | antisense（5'-3'） |
| *LPP1* (homo-830) | GCCUCACUUCUUGGAUGUUTT | AACAUCCAAGAAGUGAGGCTT |
| *LPP1 (*homo-627) | GGUGGAAUAAUCAUUCCAUTT | AUGGAAUGAUUAUUCCACCTT |
| *LPP1* (homo-484) | UCGAUGUGCUCUGCGUGUUTT | AACACGCAGAGCACAUCGATT |
| *PCYT1A* (homo-434) | GCGACCUGUGAGAGUUUAUTT | AUAAACUCUCACAGGUCGCTT |
| *PCYT1A* (homo-774) | GCAGGCAUGUUUGCUCCAATT | UUGGAGCAAACAUGCCUGCTT |
| *PCYT1A* (homo-1082) | GGAAAUGUUUGGUCCGGAATT | UUCCGGACCAAACAUUUCCTT |
| Negative control | UUCUCCGAACGUGUCACGUTT | ACGUGACACGUUCGGAGAATT |
